# Learning Performance in Adaptive Learning Systems: A Case Study of Web Programming Learning Recommendations

**DOI:** 10.3389/fpsyg.2022.770637

**Published:** 2022-01-28

**Authors:** Hsiao-Chi Ling, Hsiu-Sen Chiang

**Affiliations:** ^1^Department of Marketing, Kainan University, Taoyuan, Taiwan; ^2^Department of Information Management, National Taichung University of Science and Technology, Taichung, Taiwan

**Keywords:** computer programming, personality traits, personalization, learning motivation, decision tree

## Abstract

Students often face challenges while learning computer programming because programming languages’ logic and visual presentations differ from human thought processes. If the course content does not closely match learners’ skill level, the learner cannot follow the learning process, resulting in frustration, low learning motivation, or abandonment. This research proposes a web programming learning recommendation system to provide students with personalized guidance and step-by-step learning planning. The system contains front-end and back-end web development instructions. It can create personalized learning paths to help learners achieve a sense of accomplishment. The system can help learners build self-confidence and improve learning effectiveness. In study 1, the recommendation system was developed based on the personal data and feedback of 41 professional web design engineers. The system uses C4.5 decision tree methods to develop a programming learning recommendation model to provide appropriate learning recommendations and establish personalized learning paths. The test group included 13 beginner programmers. After 4 weeks’ programming instructions in front-end and back-end web development, the learners were interviewed to understand their preferences and learning effectiveness. The results show that the effectiveness of the recommendation system is acceptable. In study 2, online real-time feedback and adaptive instruction platform is developed, which is different from the past adaptive curriculums mainly using the Internet platform and only the submitted assignments to determine the newly recommended learning process for students. The study found that the students’ learning performance in the adaptive instruction group is better than those in the fixed instruction group.

## Introduction

Computer programming courses are popular and attracting much attention in recent years. Many schools begin to teach the students in different age computer language. However, programming logic, visualizations, and language expressions are difficult to understand. The mismatch between programming conventions and human thought processes results in students frequently becoming frustrated and losing self-confidence in the study of computer programming. In addition, the mismatch between course difficulty and learner ability will cause the learner to fall behind, giving rise to considerable anxiety and frustration, which can also result in learner attrition. To effectively solve learner frustration and anxiety, students should ideally be provided with courses of instruction with carefully graduated levels of complexity, allowing students to achieve a sense of accomplishment which, in turn, will drive increased learning motivation.

In order to enhance students’ learning motivation and decrease their frustration in the learning process, many companies are developing customized learning systems. Although the learning effects after using the customized learning systems have been widely discussed, few studies examined the learning effects in personalized web programming learning-oriented recommendation systems. Hence, in study 1, this research proposed designing a personalized and learning-oriented web programming recommendation system to recommend suitable programming instruction. Personalized instruction can reduce frustration for novice learners and help them maintain positive learning motivation. In study 2, an experimental design is used to test the effect of an adaptive instruction platform on the learning outcome of HTML/CSS programming. The system in study 1 can help students understand which learning path is the most suitable for them, while study 2 will verify students’ learning performance on the adaptive instruction platform. The results and findings can provide helpful insights for web programming course design.

## Literature Review

### Adaptive Instruction

Adaptive instruction can be defined as adjusting instructional plans provided by instructors in their instructional process according to students’ learning differences. In other words, adaptive instruction can provide a personalized learning process according to students’ needs (Wang et al., 2020). When using adaptive instruction, instructors have to design and revise their instructional plans according to students’ differences and learning progress and modify their instructional modes to cope with the diverse needs of individual students. More specifically, adaptive instruction is also known as differentiated instruction, during which students would learn better when instructors proactively address their differences and learning gaps (Smit and Humpert, 2012).

Adaptive instruction can be used in personalized learning platforms. Past research adopted adaptive instruction in computer programming courses in an online environment and evaluated students’ learning performance (Si et al., 2014). The results found that the students’ learning performance in the adaptive instruction group is better than those in the fixed instruction group. Recent studies also show that adaptive teaching competency significantly affects students’ achievement (Brühwiler and Vogt, 2020). Hence, adaptive instruction/teaching has been demonstrated to be a helpful teaching method (Ankrum et al., 2020; Iterbeke et al., 2021).

### Learning Motivation and Learning Outcome

Programming language is a subject that emphasizes logic and practice, where many studies have shown that the assessment of learning outcomes in a programming language cannot be based solely on logic tests. The assessment via implementing practical programs also must be considered in assessing students’ abilities (Lau and Yuen, 2011; Yang et al., 2015). As a result, in this study, the learning outcome assessment of programming is realized in terms of both the practice-based assessment and grading by teaching assistants (TAs) (Wang et al., 2012; Yang et al., 2015). Past studies show that students’ motivation will influence learning outcomes (Law et al., 2019; Ling et al., 2020; Chen and Hsu, 2022). Adaptive instruction can attract students’ attention and enhance their learning motivation and outcome. Hence, personalized learning platforms can increase students’ learning performance in computer programming courses.

## Study 1

### Research Method

A questionnaire was distributed to professional web programmers working in the information engineering industry to collect a training data set. Then, we used the data and a decision tree method to construct a learning path recommendation model to provide programming students with recommendations for learning content (front-end and back-end web programming) suitable to their learning level, along with related course content and learning units. Feedback from test subjects at the end of the research is then used to improve recommendation model performance incrementally.

#### Research Tools

##### Questionnaire

The data collected by the questionnaire was used to develop recommendations for appropriate learning paths based on learner personality traits, logical thinking skills, and degree of dispositional resilience. Learning outcomes are related to personality traits (O’Connor and Paunonen, 2007), and personality traits also help determine learner aptitude for different subjects (Zhang and Ziegler, 2016). The present study used Saucier (1994) concept of “Mini-Marker” to compile the questionnaire to measure five major personality traits (see Supplementary Appendix A). Following Bosley (2013), the Logical Competence section posed questions designed to assess the subject’s logical reasoning skills, with each of 20 questions worth 5 points, for a maximum score of 100 points. The Dispositional Resilience section was designed using Bartone (1995) Dispositional Resilience Scale-15 (DRS-15).

##### Course Design

The learning path design includes a step-by-step learning unit progression, checkpoints, and teacher assessment. Each learning unit uses corresponding exercises as checkpoints to evaluate learning effectiveness.

#### Decision Tree

Decision Trees are a classification method that can create a model to predict results by decision rules. Decision Trees in this study use the C4.5 classification algorithm to generate decision rules. The rules in this study include students’ personality traits, gender, stress resilience, and logical thinking ability.

#### Construction of a Learning Path Recommendation Model

##### Data Collection

The questionnaire presented in Table 1 was used to collect data over 1 month. A total of 41 valid responses were received, including 31 males and ten females, with an age range of 22–40 years old. All respondents worked as professional programming engineers in eight different information engineering companies, working on front-end or back-end web applications.

**TABLE 1:** Learning path recommendation evaluation.

No.	Recommendation path	Learning outcome	Accuracy
1	Back-end	Good	True
2	Back-end	Good	True
3	Back-end	Normal	True
4	Back-end	Good	True
5	Back-end	Bad	False
6	Back-end	Normal	True
7	Back-end	Bad	False
8	Front-end	Bad	False
9	Front-end	Normal	True
10	Front-end	Good	True
11	Back-end	Normal	True
12	Front-end	Bad	False
13	Front-end	Good	True

##### Decision Tree Model Building

Following the data collection results, gender, personality characteristics, logical thinking capacity, and stress resilience are taken as independent variables, with front-end and back-end web programming learning performance used as the dependent variables. The C4.5 decision tree algorithm was applied to the training data set to construct a learning path recommendation model. Preliminary analysis results suggest that more open personality traits and males with moderate to high-stress resilience are better suited to back-end programming. In contrast, front-end programming is better suited to those with more complex personality traits and stronger logical reasoning capability.

### Results and Discussion

In order to evaluate the effectiveness of the learning path recommendation model, this study convened a testing group of 13 Asset Management majors from a university in Taiwan, including eight males and five females. All test subjects were novice programmers. At the beginning of the course, we collected data on personality traits, stress resilience, and logical thinking capabilities through the questionnaire. The results were then used to provide recommendations for suitable learning concepts and paths for a subsequent 4-week learning program on front-end and back-end web programming design. Learning checkpoints and expert evaluation during and after this study were used to evaluate the learning effectiveness of the program, followed by interviews to assess subject perceptions of their recommended learning path and its compatibility with their learning interests. We thus attempted to verify the accuracy of the inference model for the decision tree-constructed learning path recommendation program.

Actual results matched the recommended results for 9 of the 13 test subjects, for a recommendation accuracy rate of 69.23%, indicating that the proposed system can provide accurate programming learning paths for some learners. However, the model requires considerable refinements using large volumes of learner data. The establishment of the model and prototype system achieves the goals of providing personalized and adaptive programming learning recommendations. Post-completion interviews with test subjects provided the following findings:

(1) Model recommendation accuracy was 69.23%, and interview results point to key factors for inaccurate recommendations, particularly individual learning status in terms of lack of learning motivation and inability to focus on the learning process. Aside from sub-optimal learning guidance results, subjects reported that the learning path recommendation and content design did not make a strong impression.
(2) Test subjects were found to be broadly diverse in terms of personality traits, stress resilience, logical reasoning capability, self-confidence, a propensity to frustration, diligence, and engagement.
(3) Learning performance results suggest that recommendation of an appropriate learning path does not guarantee good learning performance because test subjects differ in terms of self-expectations and diligence. Learning performance will impact learning motivation in that a sense of accomplishment will impact the learner’s subjective effect. For example, the subject performing well on front-end programming may translate the resulting sense of achievement into a preference for front-end programming, resulting in incorrect path recommendations.

## Study 2

### Adaptive Learning Platform and Instruction Content Design

In this study, an Online Real-time Feedback Adaptive Instruction Platform is constructed with the features of learning path design, adaptive recommendation, checkpoint mechanism, and timely feedback and assistance. The program design learning units are arranged according to the level of difficulty of program design, on which learning can be realized progressively step by step through the design of interactive guided learning.

1. Learning Path Design:
In this study, three learning units with different levels of difficulty for front-end program design together with corresponding implementation examples as an assessment mechanism to form the three learning paths with different levels of difficulty, each of which will have 17 checkpoints of level and be presented on the platform allowing students to learn through the platform.
2. Adaptive Recommendation:
In this study, adaptive recommendation of learning paths based on students’ personality traits, program design ability, logic capability, and stress resistance is made. The personality trait questionnaire and learning interest will be used as the assessment tool. Regarding program design ability, the naming of essential variables and the fundamentals such as data types are the basic knowledge. In the aspect of logic capability, the logical reasoning website^1^ has ten questions in total (one point for each question with correct answer, and ten points in total) as the assessment tool to evaluate the testees’ capability of logical reasoning. In the aspect of stress resistance, the learning pressure questionnaire is used as the assessment tool.
3. Checkpoint Mechanism:
Each learning unit is designed with practice implementation assessment questions with 17 levels to serve as checkpoints. In addition to the learner’s score for completing the practice questions, the learner will be graded by a teaching assistant (TA) based on his/her logic and structure written for the program. The practice implementation exercises involve 50% of the entire score grading, and the remaining 50% comes from the score grading given by TAs. Meanwhile, a threshold will be set to determine whether the student has truly learned the core concepts of the learning unit. Only those who pass the checkpoints can move on to the next level of learning, thereby learning step by step for the subsequent learning levels. Only those who have passed the comprehensive score threshold will pass the level’s checkpoints to ensure that their learning outcomes are met before moving on to the next level. Observing the levels of checkpoints and learning duration helps instructors keep track of their students’ learning situations.
4. Timely Feedback and Assistance:
Four TAs are assigned to the curriculum design of each learning path. If any student stays at a particular level of a learning unit for too long or his/her TA finds that the student’s idea is in confusion upon reviewing his/her program logic, the TA will ask about the student’s learning situation and give him/her assistance to reduce the chance of the student’s giving up due to frustration.

### Research Subjects

The target subjects of this study are information management majors of a university of science and technology night division, who agree to join this study by filling in the consent forms before the experimental study proceeds. The students are divided into two groups. The experimental and control groups were first given program design instruction in the computer lab. The experimental group students then used the Online Adaptive Instruction Platform to practice program design exercises. At the same time, the control group students used the computers in the computer lab for program design practice exercises instead. After collecting their learning outcome using questionnaires, an independent sample *t*-test was used to compare the experimental and control groups’ post-test scores.

### Experimental Design: Adaptive Instruction

Prior to the experiment, the testees are required to undergo a pre-test assessment and a logic test assessment in order to understand the students’ previous programming learning experience. The testees are further divided into an experimental group and a control group depending on whether adaptive instruction together with the “Online Real-time Feedback Adaptive Instruction Platform” is given or not. During the 16-week course, the instructor would instruct the course by following the course content schedule as it has been arranged, and at the end of each week’s class, a class assignment is designed that includes a number of concept designs delivered in the class. Students in both groups are required to practice at home and hand in assignments to determine their learning outcomes.

#### Experimental Flow

**FIGURE 1:**
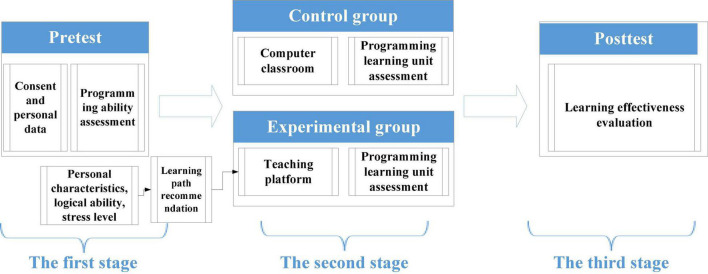
Experimental procedure flow chart.

The experiment process was divided into three stages, and the total duration was 16 weeks, as shown in Figure 1. A pre-test was first given in the first stage to ensure no significant difference in the programming ability between two groups of testees before giving the experiment.

In the second stage, both the experimental group and the control group were given the computer hands-on HTML + CSS program design course at the computer lab. The students in the experimental group used the Online Adaptive Instruction Platform for conducting practice implementation exercises. The students in the control group used the off-line computer practice exercise method, typically found in traditional program design instruction. After each week’s class, students were required to submit weekly program assignments to record the learning progress of the two groups of students. The adaptive learning content is thereby adjusted, and the TA-assisted instruction is timely given according to the grading status of each submitted assignment.

During the second stage, a midterm project was given to students for assessment in the ninth week, and students were also required to fill in the learning motivation questionnaires to assess their midterm learning outcomes and motivation.

A post-test was conducted in the third stage by giving students a final term project to assess their learning outcomes. The students were also required to fill in the learning motivation questionnaires to assess their final term learning outcomes and motivation.

### Learning Outcome Assessment

Both groups of testees were given the curriculum with the same instruction syllabus. For the experimental group students, the course content was mainly adjusted according to their individual learning situations such that the final learning progress of each student turned out to be different. Consequently, their learning outcomes were not assessed by their final assignments submitted, but the exercises are given at each class session as the basis of assessment. The way of learning outcome assessment method is divided into two parts:

#### Completion of Practice Implementation Assignments Given

The assignments should be completed to the specified level; for each assignment, there are10–15 specific requirements to be completed, and students have to fulfill the requirements to get the grade points; the completion of practice implementation assignment takes up 50% of the entire grading score.

#### Program Structure

The learning of HTML and CSS does not need strong program logic but needs the ability of design and aesthetics. The scoring of this part is performed by TAs, which accounts for 50% of the entire grading score.

### Experimental Results

**FIGURE 2:**
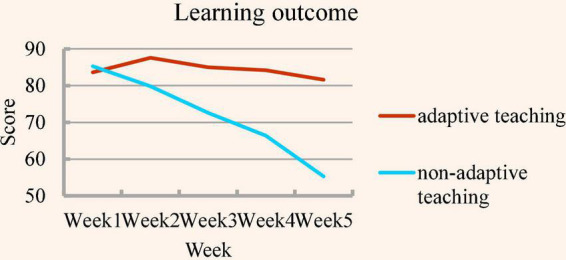
Learning outcome comparison chart (5 weeks).

In the experiment, the mean grading scores of their take-home practice implementation assignments after each class session were plotted as a trend graph. The 5 weeks of learning outcome as shown in Figure 2. Figure 2 shows that the learning outcome of the experimental group stays at a certain level; on the contrary, the learning outcome of the control group decreases as the course progresses. The experimental group testees were able to ensure that they had learned the prior knowledge before proceeding to advanced learning. In contrast, the control group testees were forced to move on to the next stage after understanding only a portion of the program design concepts. They, therefore, could not maintain the same learning outcome as that of the experimental group.

## Conclusion

This research used professional web programmers’ gender, personality traits, stress resilience, and logical reasoning capabilities in the decision tree C4.5 algorithm to develop a personalized web programming learning-oriented recommendation platform to generate personalized learning paths optimally suited for individual learners. The platform can enhance learning motivation and effectiveness for novice learners. Past research results suggest that, in addition to the above characteristics, learning outcomes would be influenced by other factors such as learning attitude, self-confidence, and frustration.

Experimental results show that the developed system fulfills basic requirements, and some subjects felt the recommendation system helped match them with an appropriate learning program. Improvements to the training sample can increase system accuracy and performance. The present study is limited by the small sample size, making it difficult to quantify each variable’s impact effectively. Future work will focus on improving recommendation accuracy by increasing the sample size and improving the feedback mechanism. Moreover, in this study, only two types of computer programming languages are tested. If the learning recommendation systems can recommend more different learning content, the learning outcome and the accuracy will be better.

In this project, based on the educational theory of adaptive instruction as the philosophy, an online learning platform and adaptive course content with corresponding assessment mechanisms (checkpoints) are developed to form a learning path through the progressive step-by-step design of learning units. In addition, an online learning feedback platform together with TAs’ timely assistance to help resolve the difficulties encountered by students while learning program design with the instructor, providing a solution to the problems in program design learning and instruction. The study results found that adaptive instruction can better control students’ learning situations, identify students’ problems early and provide timely and appropriate assistance to help improve students’ diverse levels and qualities. Moreover, the study results also indicate that assistance was given by TAs and online teaching platforms allow more resources to fulfill adaptive instruction’s ideas.

The limitations of this study should be mentioned. First, the sample size is relatively small. The effectiveness of the system may be different in different student groups. Second, the target subjects in this study were the students in central Taiwan. The generalizability of the findings needs further verification. Third, the course in this study is short. More analyses are needed in longer courses.

## Data Availability Statement

The raw data supporting the conclusions of this article will be made available by the authors, without undue reservation.

## Author Contributions

H-CL contributed to the research topic and the methodology. H-SC contributed to the experimental design and results. Both authors contributed to the article and approved the submitted version.

## Conflict of Interest

The authors declare that the research was conducted in the absence of any commercial or financial relationships that could be construed as a potential conflict of interest.

## Publisher’s Note

All claims expressed in this article are solely those of the authors and do not necessarily represent those of their affiliated organizations, or those of the publisher, the editors and the reviewers. Any product that may be evaluated in this article, or claim that may be made by its manufacturer, is not guaranteed or endorsed by the publisher.

## Footnotes

1
https://www.123test.com/logical-reasoning-test
