# Supplementary material for: Learning Performance in Adaptive Learning Systems: A Case Study of Web Programming Learning Recommendations
Source: Front Psychol. 2022 Jan 28;13:770637. doi: 10.3389/fpsyg.2022.770637 (PMC8831801; doi:10.3389/fpsyg.2022.770637)

APPENDIX A

QUESTIONNAIRE QUESTIONS

Constructs	Factors	Item
Personality traits	Openness to experience	I react differently to others when confronting problems.<br>I can use different concepts/objects to produce new concepts/objects.<br>I like to explore philosophical concepts.<br>I can quickly get the hang of new things.<br>I don't like to let simple things get complicated.<br>I try to explore the deeper meanings of events.
	Conscientiousness	I can properly allocate resources to complete tasks.<br>I usually complete my work/homework ahead of schedule.<br>I approach my work/homework by first thinking about the overall problem and steps to completion.<br>I'm not a perfectionist, I prefer to just get things done.<br>I don't think too much about the future. I prefer to live in the moment.<br>I don't focus on small details while doing things.
Personality traits	Extraversion	I like to talk to people, and I'm good at keeping a conversation going.<br>I like to participate in various social events.<br>I like risks and challenges, regardless of the outcome.<br>I feel uncomfortable when I'm exposed to new people or environments.<br>I am used to feeling alone.<br>Even without a full night's sleep, I can still face the coming day with a lot of energy.
	Agreeableness	I feel empathy for the misfortune of others.<br>I am willing to make sacrifices to help others.<br>I try to respect and cooperate with people who disagree with me.<br>I keep a cool head under pressure.<br>I forgive easily, even when people make trouble for me.<br>I don't like to admit being wrong about something.
	Neuroticism	I worry about bad things that might happen to me.<br>I am suspicious that people who are kind to me have ulterior motives.<br>I attribute the success of others mostly to good luck.<br>I prefer to be myself, and don't care about what others think.<br>My mood is easily affected by other people.<br>I let the things other people say get to me.
Dispositional Resilience	Commitment	Most of my life is spent doing things that are meaningful.<br>I feel that my life is somewhat empty of meaning.<br>I really look forward to my work.<br>Most days, life is really interesting and exciting for me.<br>Life in general is boring for me.
	Control	By working hard you can nearly always achieve your goals.<br>How things go in my life depends on my own actions.

Constructs	Factors	Item
		I don't think there's much I can do to influence my own future.<br>It is up to me to decide how the rest of my life will be.<br>My choices make a real difference in how things turn out in the end.
	Challenge	I don't like to make changes to my regular activities.<br>I enjoy changing up my routine.<br>It bothers me when my daily routine gets interrupted.<br>I enjoy the challenge of doing more than one thing at a time.<br>I like having a daily schedule that doesn’t change very much.
Logical thinking		Please identify the missing number: 9、11、8、10、7、?、6<br>Please identify the missing number 1、1、2、3、5、8、?<br>Please identify the missing number: 2、3、5、7、?、13.....、103<br>Please identify the missing number: 1、2、4、7、11、?<br>One of the following words does not fit the category: bear, rabbit, eagle, wolf, fox<br>One of the following words does not fit the category: pizza scissors, fork, spoon, hammer, chopsticks<br>One of the following words does not fit the category: shark, whale, crocodile, octopus, squid<br>Select an image from the lower row to complete the sequence in the upper row.<br>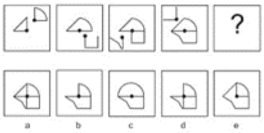<br>Select an image from the lower row to complete the sequence in the upper row.<br>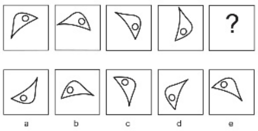
		Select an image from the lower row to complete the sequence in the upper row.<br>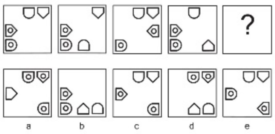<br>When unfolded, which of the cubes matches the unfolded cube to the right?<br>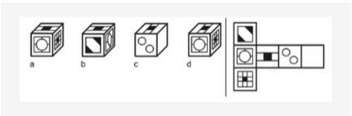<br>When unfolded, which of the cubes matches the unfolded cube to the right?<br>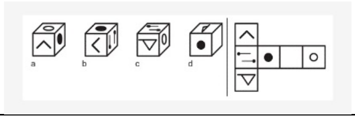
Logical thinking		“Each plant has a window, and every window has a letter, thus each plant has a letter.” Is this statement logically true or false?<br>“The water bottle can dance, but cannot swim. The bag can swim, but cannot dance. The chair can swim and dance. Inference: the chair is smarter than the water bottle and the bag.” Is this statement logically true or false?<br>“All computers listen to music. Music can sit, so computers can sit.” Is this statement logically true or false?

Constructs	Factors	Item
		“There are three people: A, B and C. A says B or C is lying. B says A is lying. C says both A and B are lying.” Which A, B, C, or B+C is telling the truth?
		Use the logical relationship between the numbers to complete the pie chart:

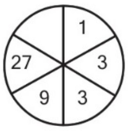

Use the logical relationship between the numbers to complete the pie chart:

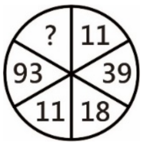

Use the logical relationship between the numbers to complete the grid:

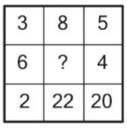

Use the logical relationship between the numbers to complete the grid:

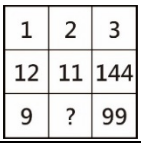

Supplement: Supplementary file 1 [file Data_Sheet_1.pdf]
